# A Novel Approach to Microfibrillated Cellulose Modification for Use as a Filler in ENR-Based Compounds

**DOI:** 10.3390/polym18172128

**Published:** 2026-08-31

**Authors:** Andrea Bernardi, Auke Gerardus Talma, Nick Helthuis, Anke Blume

**Affiliations:** 1 Department of Elastomer Technology and Engineering, University of Twente, Drienerlolaan 5, 7522 NB Enschede, The Netherlands; a.g.talma-1@utwente.nl (A.G.T.); a.blume@utwente.nl (A.B.); 2 Production Technology, Faculty of Engineering Technology, University of Twente, Drienerlolaan 5, 7522 NB Enschede, The Netherlands; n.g.j.helthuis@utwente.nl

**Keywords:** Microfibrillated cellulose, epoxidised natural rubber, sustainable filler, bio-based raw materials, histidine, vulcanisation, carbon black, Payne effect decrease, SEM characterisation, polymer–filler interaction

## Abstract

In the last few years, the tyre industry has faced new sustainability challenges, mainly regarding the substitution of fossil-based ingredients with bio-based raw materials. In fact, most of the largest tyre companies worldwide have publicly declared the objective of producing tyres with 100% sustainable materials by 2050. The main ingredients in a tyre compound are the polymer matrix and the reinforcing filler, but while natural rubber (NR) already represents a well-established bio-based alternative to synthetic polymers, the replacement of conventional reinforcing fillers remains a significant challenge. In fact, carbon black (CB), a fossil-based raw material produced from petroleum-derived feedstock, is still the main filler used in rubber compounds worldwide. A promising candidate for its replacement could be Microfibrillated cellulose (MFC): a bio-based, biocompatible, renewable, and non-toxic material, also obtained from waste biomass, with a lower density and a higher surface reactivity with respect to CB. However, the polar functional groups on its surface make it extremely incompatible with the non-polar rubber matrices used for tyre formulations. To overcome this limitation, effective compatibility strategies are required to exploit and boost these surface functionalities and promote the formation of a novel filler–polymer network. In this work, a new approach for MFC functionalisation is developed, and the synthesis and characterisation of the modified material are reported. This strategy is further applied to develop innovative MFC-reinforced epoxidised natural rubber (ENR) compounds, whose properties are compared to conventional CB-filled systems.

## 1. Introduction

Cellulose is the most abundant natural polymer on Earth: it is the main constituent of plant and wood cell walls, together with hemicellulose and lignin, and is also present in bacteria, fungi, algae, and even animals (e.g., tunicates) [1].

Despite its use dating back centuries [2], the cellulose production industry has experienced significant growth [3,4] in the past decade, driven by increasing demand for sustainable materials, technological advancements, and evolving market trends. Innovations include the production of cellulose nanoparticles and cellulose nanocomposites, which have opened new avenues for applications in fields such as biomedical engineering, electronics, and environmental technology [5,6,7,8,9,10,11]. Cellulose nanomaterials, also called “nanocellulose”, are in fact one of the few examples of commercially available nanoparticles derived from sustainable natural resources.

This unique feature has recently attracted the interest of the tyre industry as well. In fact, sustainability has become a key concern also in the tyre sector, which is currently facing new challenges, especially regarding the replacement of fossil-based raw materials. Recently, leading tyre manufacturers have publicly outlined their strict timelines and strategies for integrating sustainable raw materials, such as recycled and bio-derived components, into their tyre products [12,13,14,15,16]. Their goals are challenging and ambitious, as most of them aim to reach at least 40% sustainable raw materials in tyre products by 2030, growing to 100% by 2050. In this regard, cellulose, especially in its nanometric forms, has great potential to be used as a sustainable filler in tyres, given its bio-derived nature and suitable particle dimensions. In fact, the main challenge for sustainable raw materials (e.g., lignin, recycled carbon black) is the reinforcing effect [17], since they are all considered as low or semi-reinforcing fillers, and they cannot fully substitute highly reinforcing CB, a fossil-based raw material still widely used in tyre compounds [18,19,20]. In this context, Microfibrillated cellulose (MFC), with a typical diameter of 10–100 nm, a typical length of 0.5–10 µm, and a rope-like structure [21,22,23,24], seems to have suitable characteristics to serve as a reinforcing filler in tyre compounds [18,19,20].

However, the physicochemical characteristics of the two materials are completely different [25,26,27,28]. In fact, CB is an apolar filler, composed mainly of carbon and hydrogen, with controlled particle size, surface area, and porosity. It is specifically designed to be extremely compatible with the apolar polymer matrices used in tyre compounds, and the reinforcing effect is based mainly on physical interactions and chemical affinity [29,30]. By contrast, nanocellulose is a strongly polar polymer composed of glucose monomers, with the chemical formula (C_6_H_10_O_5_)_n_. Its surface bears three hydroxyl groups per repeating unit [31,32], leading to a significant polarity mismatch and chemical diversity with commonly used rubber matrices. Moreover, these hydroxyl groups are poorly reactive and hardly accessible, since they are coordinated via both intra- and intermolecular H-bonding to the crystalline structure of cellulose [33,34]. Therefore, the reinforcing effect of MFC cannot be purely physical, as for CB, but must rely on positive chemical interactions.

The most widely used strategy for increasing MFC compatibility with rubbers is to modify the nanocellulose surface with apolar functionalities to improve compatibility with the rubber matrix and, therefore, dispersion [24,27,28,35,36]. However, this approach still relies on physical interactions between the modified (hydrophobised) nanocellulose and the rubber, so the reinforcing effect remains limited due to the absence of porosity and the limited slippage at the interface between polymer chains and filler particles.

Another approach involves pre-dispersing nanocellulose in a latex elastomer to create a masterbatch that, after coagulation and water removal, is blended with other elastomers in a second compounding step. Latexes typically used are NR, SBR, NBR, HNBR and ENR [24,37,38,39,40]. However, even if this strategy can improve filler dispersion, the reinforcing mechanism still relies on physical interactions, with all the limitations mentioned above.

One last approach is to use a chemically modified polymer bearing polar groups in the main chain to improve compatibility with nanocellulose. In particular, epoxidised natural rubber (ENR) has recently been used to produce cellulose-filled compounds, achieving improved filler dispersion [37,41,42,43,44,45]. Nevertheless, the reinforcing mechanism of the filler still relies on physical interactions.

A different strategy involves the use of a coupling agent to chemically bond nanocellulose to the polymeric chains [36,46,47,48,49,50], similarly to the silica–silane approach, widely used in the tyre industry for decades [51,52]. In this way, the polymer–filler compatibility would be optimal. However, the surface reactivity of nanocellulose is worse than silica, since the C-OH groups are less reactive than the Si-OH, and therefore a suitable coupling agent for nanocellulose has not yet been discovered.

This research follows a different approach, combining the use of a polar rubber like ENR with the exploitation of the surface chemistry of cellulose. In fact, cellulose reactivity towards epoxides is well known in the literature [53,54], but it has never been exploited to create a polymer–filler network between nanocellulose and partly epoxidised natural rubber. The idea is to enhance this reactivity through a bio-based catalyst, which promotes and accelerates the epoxide ring opening of ENR and the subsequent reaction with the hydroxyl groups on the cellulose surface, thereby producing a strong and stable polymer–filler network.

To assess the feasibility of this approach, model reactions were first performed. These reactions were aimed at testing the reactivity of MFC towards both small (model compound) and large (bulky) epoxide-containing chemicals, and at investigating the use of a sustainable catalyst to enhance this reactivity. Once the reactivity was established and the reaction conditions were defined, these were exploited to produce ENR-based MFC-filled rubber compounds. This approach has already been tried in the literature, and a certain reactivity between cellulose and ENR has already been hypothesised [37,41,43,55,56]. Nevertheless, an ENR-based compound filled with only nanocellulose in such a high amount (ca. 25 *w*/*w*%) represents a novelty, since usually other fillers were utilised in combination with nanocellulose, or the compound was blended with other rubber matrices and used in small amounts [24,27,28,45]. Moreover, since these compounds are intended for the tyre industry, both MFC and the catalyst were added *in situ*, without introducing any additional mixing step.

This research work is therefore divided into two parts. In the first part, model reactions between the MFC and epoxide-containing chemicals are described, along with characterisation data obtained from the modified MFC (FTIR, TGA, and SEM images). In the second part, the reactivity of MFC towards epoxide functional groups is exploited to produce rubber compounds based on ENR, filled with 40 phr of MFC. The properties of these compounds are compared with those of a reference compound containing 40 phr of a reinforcing grade CB (N330), in terms of cure behaviour, Payne effect, hardness and tensile properties. In addition, SEM images of the two compounds in the presence of the catalyst are also reported, to elucidate the filler dispersion inside the polymer matrix.

To the best of our knowledge, this is the first time such a high amount of MFC has been successfully incorporated as a filler in a rubber compound.

## 2. Materials and Methods

### 2.1. Materials

The rubber matrix used in this work was epoxidised natural rubber (ENR) with a nominal epoxide content of 50 mol%, denoted as ENR-50, supplied by Muang Mai Guthrie Public Co., Ltd., Phuket, Thailand.

The fillers used were two commercial grades of Microfibrillated cellulose (MFC) and one reinforcing grade of carbon black. The MFC samples were Exilva F (viscosity in water > 20,000 mPas, pH = 5–7), and Exilva P (viscosity in water > 14,000 mPas, pH = 5–7), both kindly provided by Borregaard AS, Sarpsborg, Norway. MFC samples were in two different water concentrations: for the model reactions, Exilva (F or P)-L (2 *w*/*w*% suspension), with solid content 1.7–2.1 *w*/*w*%, was used, while for rubber compounds, Exilva (F or P)-V (10 *w*/*w*% paste), with solid content 9–11 *w*/*w*%, was used.

Carbon black used was VULCAN^®^ 3 (conforming to ASTM grade N330) from Cabot Corporation, Boston, MA, USA, with declared STSA surface area of 76 m^2^/g, Iodine number of 82 mg/g and oil adsorption number (OAN) of 102 mL/100g. L-histidine (≥99%) used as catalyst was purchased from Sigma-Aldrich, St. Louis, MO, USA, and used as received.

Other ingredients for the model reaction were epoxidised soybean oil (ESO) Lankroflex E2307, kindly provided by Valtris Speciality Chemicals (Manchester, UK), with an oxirane oxygen value of 6.6 *w*/*w*%, and a renewable carbon content of 100%; and allyl glycidyl ether (AGE, ≥99%), purchased from Sigma-Aldrich. Both products were used as received. Ethanol (100%) was used as solvent, purchased from Boom B. V. (Meppel, The Netherlands) and used as received.

Other ingredients used for rubber compounds were as follows: Zinc oxide (ZnO) and stearic acid as activators were purchased from Millipore Sigma, Hamburg, Germany; the cure package was composed of sulphur and N-tert-butyl-2-benzothiazolesulfenamide (TBBS), both from Caldic B.V., Rotterdam, The Netherlands; antioxidant N-(1,3-dimethylbutyl)-N′-phenyl-p-phenylenediamine (6-PPD) was purchased from Flexsys, Chemicals Belgium NV, Antwerp, Belgium.

### 2.2. Synthesis of Modified MFC

As mentioned above, model reactions are first performed to test the reactivity of MFC towards epoxide-containing molecules, and to assess the reaction conditions which will be used for compound production.

#### 2.2.1. Reaction Between MFC and AGE

In a 500 mL beaker equipped with a mechanical stirrer, 100 g of Exilva (P or F)-L (2 *w*/*w*% in water, corresponding to ca. 2 g of dried material, 1.23 × 10^−2^ mol) were poured. Then, 200 mg of histidine (10 *w*/*w*% with respect to dried MFC) were added, together with 5 mL of deionised water. The mixture was placed in an oil bath at T = 140 °C, under mechanical stirring (ca. 250 rpm). After 1 min, 2 mL of allyl glycidyl ether (AGE, 1.69 × 10^−2^ mol, in excess with respect to MFC) were added inside the beaker, together with 5 mL of ethanol. The reaction was carried out for 30 min (T = 140 °C, 250 rpm). At the end, the product (almost dried) was collected in a Gooch crucible over a vacuum flask, then abundantly washed with deionised water (3 × 100 mL) and ethanol (3 × 100 mL) and filtered under reduced pressure. At the end, the product was dried at room temperature in air for around 3 days. It was finally recovered as a yellowish fine powder.

#### 2.2.2. Reaction Between MFC and ESO

In a 500 mL beaker equipped with a mechanical stirrer, 100 g of Exilva (P or F)-L (2 *w*/*w*% in water, corresponding to ca. 2 g of dried material, 1.23 × 10^−2^ mol) were poured. Then, 200 mg of histidine (10 *w*/*w*% with respect to dried MFC) were added, together with 5 mL of deionised water. The mixture was placed in an oil bath at T = 140 °C, under mechanical stirring (ca. 250 rpm). After 1 min, 2 g of epoxidised soybean oil (ESO, 1:1 by weight with respect to dried MFC, ca. 2 × 10^−3^ mol) were added inside the beaker, together with 5 mL of ethanol. The reaction was carried out for 30 min (T = 140 °C, 250 rpm). At the end, the product (almost dried) was collected in a Gooch crucible over a vacuum flask, then abundantly washed with deionised water (3 × 100 mL) and ethanol (3 × 100 mL) and filtered under reduced pressure. At the end, the product was dried at room temperature in air for around 3 days. It was finally recovered as a yellowish fine powder.

### 2.3. Filler Characterisation

To understand the feasibility of the reactions under the identified conditions, the modified MFC was characterised using different analytical techniques, listed below.

#### 2.3.1. Fourier Transform Infrared Spectroscopy (FTIR)

All the pristine and modified MFCs were characterised by Fourier transform infrared (FTIR) spectroscopy using a Nicolet iS50 FTIR spectrometer (Thermo Fisher Scientific, Waltham, MA, USA). The system was equipped with a built-in diamond Attenuated Total Reflection (ATR) accessory and a DLaTGS detector.

All spectra were recorded over a wavenumber range of 4000 cm^−1^ to 550 cm^−1^ with a spectral resolution of 4 cm^−1^ and an average of 16 scans per sample. All data were processed using OMNIC software (version 9.16, Thermo Fisher Scientific, Madison, WI, USA).

#### 2.3.2. Thermogravimetric Analysis (TGA)

Thermogravimetric analysis (TGA) was performed using a TGA 550 analyser (TA Instruments, New Castle, DE, USA). Prior to analysis, samples were kept under vacuum for ca. 30 min. Approximately 5–10 mg of each modified and unmodified MFC sample was placed in a tared platinum pan. The initial gas was set to nitrogen (flow rate of 20 mL/min), and the temperature program consisted of an initial equilibration step at 25 °C, followed by heating from 25 to 100 °C at a rate of 10 °C/min, then a 5 min isothermal step at 100 °C, then another heating from 100 °C to 850 °C at 10 °C/min. Subsequently, the sample is kept at 850 °C for 5 min under nitrogen, then the gas is switched to air for the last isothermal step (5 min at 850 °C).

#### 2.3.3. SEM Analysis

Scanning electron microscopy (SEM) was performed on the Exilva P-L cellulose fillers (modified and unmodified) and on MFC-filled (Exilva P-V) and CB N330-filled rubber compounds, both in the presence of histidine, using a JEOL JSM-7200F FE-SEM microscope (JEOL Ltd., Tokyo, Japan). Before microscopic analysis, the filler was fixed on a double-sided carbon tape, after which the samples were examined using a secondary electron detector at an accelerating voltage of 3 kV.

To obtain a representative cross-section of the rubber compounds, a JEOL IB-19520CCP (JEOL Ltd., Tokyo, Japan) cross-section polisher was used with an acceleration voltage of 5 kV for 8 h. Images were acquired using a secondary electron detector at acceleration voltages of 1 and 3 kV.

### 2.4. Rubber Compounds Production: Mixing Procedure

Two different kinds of compounds were prepared: one filled with 40 phr of MFC, and the other filled with 40 phr of CB N330. N330 is a reinforcing grade of CB; therefore, these compounds will be used as a reference to establish the reinforcing effect of MFC. Both grades of MFC (Exilva F-V and Exilva P-V) were tested. The effect of the catalyst was also investigated in the presence of CB (considered as an unreactive filler) and MFC (considered as a reactive filler). Therefore, a total of six compounds were produced. Their formulations are listed in Table 1 below.

The compounds were prepared using a two-stage mixing process, as outlined in Table 2 and Table 3 below. Mixing was carried out in a 50 mL Brabender Plastograph EC Plus internal mixer (Plastograph^®^ EC Plus, Brabender GmbH & Co., KG [now Anton Paar TorqueTec GmbH], Duisburg, Germany) equipped with tangential rotors (N-rotor geometry). After mixing, the compounds were sheeted on a two-roll mill (Schwabenthan Polymix 80 T, Schwabenthan Maschinenfabrik, Berlin, Germany) with a roll gap of 2 mm and a roll surface temperature of 20 °C. The sheets were subsequently stored at room temperature in a cool, dry environment for at least 24 h before the second mixing stage.

CB-filled compounds were produced in duplicate: for the first one, a standard procedure was used, which involved a non-reactive first stage at a temperature of 80 °C and a rotor speed of 50 rpm, while for the second one, the temperature of the first mixing stage was raised to 140 °C to investigate the effect of the mixing temperature on the compound’s properties. The CB-filled compound with histidine was also mixed during the first stage at 140 °C for direct comparison under the same mixing conditions. For MFC-filled compounds, the first stage included reactive mixing at T = 140 °C, variable rotor speed (not to exceed 140 °C), and repeated ram opening to ensure proper water evaporation (MFC used was Exilva-V, 10 *w*/*w*% paste in water) and to allow possible reactions to take place. In fact, the idea behind this mixing procedure was to mimic the MFC–epoxide reaction conditions previously identified through the model reactions, to test the reactivity of MFC towards the epoxide of ENR directly in situ, in the presence and in the absence of the catalyst. It is worth noting that water evaporation from the MFC produces a large amount of steam, which must be properly vented to prevent pressure buildup in the mixing chamber. Scaling up this process with a sealed-chamber mixer could be problematic, whereas an extrusion process might be preferred.

The second mixing stage was the same for both kinds of compounds.

### 2.5. Rubber Compounds Characterisation

All the produced rubber compounds were characterised using the techniques described below.

#### 2.5.1. Cure Behaviour

The vulcanisation behaviour was evaluated using a Rubber Process Analyzer (RPA, TA Instruments, New Castle, DE, USA). Cure characteristics were measured at 160 °C for 30 min at a frequency of 1.667 Hz and a strain amplitude of 6.98%, in accordance with ISO 3417. The optimum cure time (t90), minimum torque (S′min), and maximum torque (S′max) were determined from the resulting cure curves. Subsequently, the compounds were vulcanised in a Wickert laboratory press (WLP 1600/5 × 4/3) at 160 °C under a pressure of 100 bar.

#### 2.5.2. Payne Effect Measurements Method

The cured Payne effect was measured using a Rubber Process Analyzer TA Elite (TA Instruments, New Castle, DE, USA). Samples were first vulcanised in the instrument at 160 °C according to their respective t90 values. Immediately after curing, the complex shear modulus (G*) was measured at 60 °C. Strain sweep measurements were performed from 0.1% to 100% strain and subsequently from 100% to 0.1% strain at a frequency of 1.667 Hz. The second (decreasing) strain sweep was used for visualisation and data analysis.

#### 2.5.3. Tensile Test Method

Vulcanisates for tensile testing were prepared as sheets with dimensions of 90 × 90 × 2 mm^3^ using a Wickert laboratory press, cured at 160 °C for their respective optimum cure times (t90). Possible filler orientation (anisotropic effect) was not taken into account: fillers were randomly distributed inside the compounds. The cured sheets were subsequently die-cut into dumbbell-shaped specimens. Tensile tests were performed using a Zwick Z020 (ZwickRoell GmbH & Co. KG, Ulm, Germany) universal testing machine at a crosshead speed of 500 mm·min^−1^, in accordance with ISO 37 (Method A). Five specimens were tested per compound. The specimen exhibiting behaviour closest to the average response was selected for analysis and visualisation, while the results of the other four specimens were used to calculate the mean value and the standard deviations.

#### 2.5.4. Hardness Method

The hardness of the compounds was measured using a durometer Zwick 3150 hardness tester (Zwick Z05, ZwickRoell GmbH & Co. KG, Ulm, Germany) in accordance with the ISO 48-4 standard at a temperature of 23 °C. The Shore A scale was used. For each compound, three specimens were tested, and the average values were reported.

## 3. Results and Discussion

This research work is divided into two parts. In the first part, the reactivity of MFC towards epoxide functional groups is demonstrated using two different chemicals: allyl glycidyl ether (AGE), a relatively small molecule which reactivity with cellulose is already described in the literature [56,57], since it has been widely used for wood treatment [58]; and epoxidised soybean oil (ESO), a modified vegetable oil consisting of natural triglyceride molecules bearing reactive oxirane (epoxy) rings, often used as a plasticizer in combination with cellulose [59,60,61,62,63]. The reactivity of ESO towards OH groups has also been described in the literature [61], as it has been used for cellulose modification via a different synthetic approach [59,60,63]. The aim of the present study is to demonstrate that an MFC can be modified using both small (probe) molecules and bulky reactants under the same reaction conditions. Both reactions are carried out in the presence of histidine, a natural amino acid containing an imidazole ring, which is assumed to catalyse epoxide ring opening and the subsequent etherification reaction with MFC. In fact, histidine was already used as a catalyst for epoxide ring opening and subsequent reaction with phenolic OH groups (e.g., tannic acid [64] and quercetin [65]). In the present study, its catalytic effect was tested in the presence of the aliphatic OH groups of MFC. The modified MFCs were characterised using the following techniques: FTIR, TGA, and SEM analysis.

In the second part, the reactivity of MFC towards epoxide functional groups is exploited to produce MFC-filled ENR-based compounds, using pristine MFC as a starting material to try an in situ reaction with ENR epoxides. Histidine is again used to catalyse the reaction. The properties of the compounds are compared with those of a reference composite filled with CB N330. This grade of carbon black is commonly used as a reinforcing filler in tyre compounds. Moreover, CB is considered a non-reactive filler, since blank tests with histidine performed in the same reaction (and purification) conditions show no reactivity towards the two species; therefore, the comparison between CB- and MFC-filled compounds is aimed at assessing whether MFC can act as both a reinforcing and a reactive filler. In-rubber properties are investigated in terms of cure behaviour, Payne effect, and tensile properties. Also, SEM images of the MFC- and CB-filled compounds, both in the presence of histidine, are reported.

### 3.1. Model Reactions

The model reactions were carried out following the procedures described above (Section 2.2.1 and Section 2.2.2).

The reactions were performed for 30 min at 140 °C under mechanical stirring. Since MFC is already in water (2 *w*/*w*%), no additional solvent is required, except for 5 mL of ethanol, which is used to homogenise the reaction mixture and ensure the complete addition of the hydrophobic reactant (AGE or ESO). Histidine was selected as a catalyst, since it contains an imidazole ring, a carboxylic acid group and an amine group. It is considered a catalyst for cellulose etherification, since the imidazole ring is a nucleophile that can help the S_N_^2^ reaction, while the amino acid group can further enhance the reactivity by creating hydrogen bonds with the oxygen atom in the epoxide. This mechanism is also favoured by the pKa of histidine (equal to 6.5 [66]), which makes histidine act as a base or as an acid, depending on the environmental conditions.

**Figure 1:**
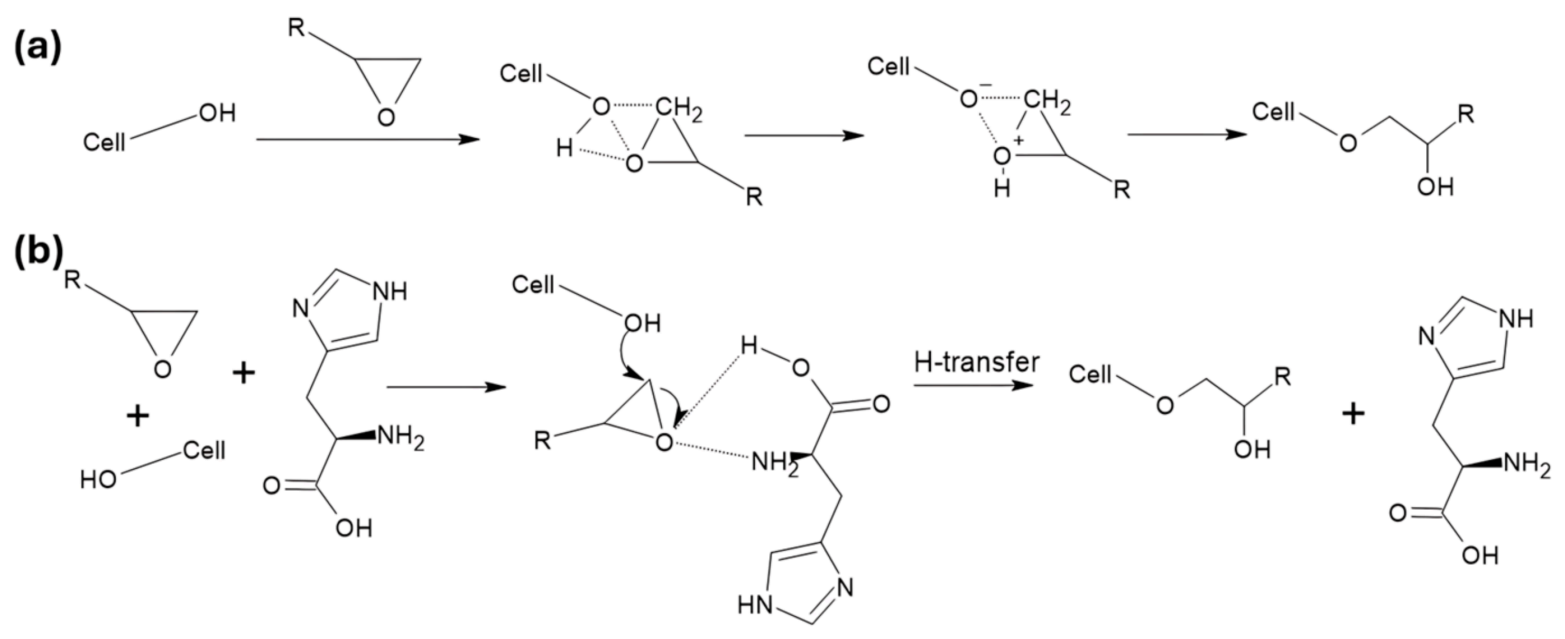
Generic mechanism for MFC–epoxide reaction: (**a**) without the catalyst, (**b**) in the presence of histidine [67,68].

A generic reaction mechanism is reported in Figure 1 below. The reaction in the presence of histidine (Figure 1b) is expressed in terms of a combination of hydrogen bonding, but it cannot be excluded that the imidazole ring is taking part in the opening of the epoxide group, as suggested in previous works [59,60,64]. Although the reactivity of the primary hydroxyl group of MFC is considered to be the highest, due to the lowest steric hindrance [67,68], at present it is not possible to clearly establish which OH group will preferentially react; therefore, a generic OH group is reported.

Blank reactions are also performed using MFC in combination with histidine to demonstrate that no reactivity is present between the two species. Reactions without histidine were not performed, since the research aims to prove the role of histidine as a catalyst, not to compare reaction kinetics. Reactant amounts and sample identifications are reported in Table 4 below.

#### 3.1.1. FTIR (ATR) Analysis

**Figure 2:**
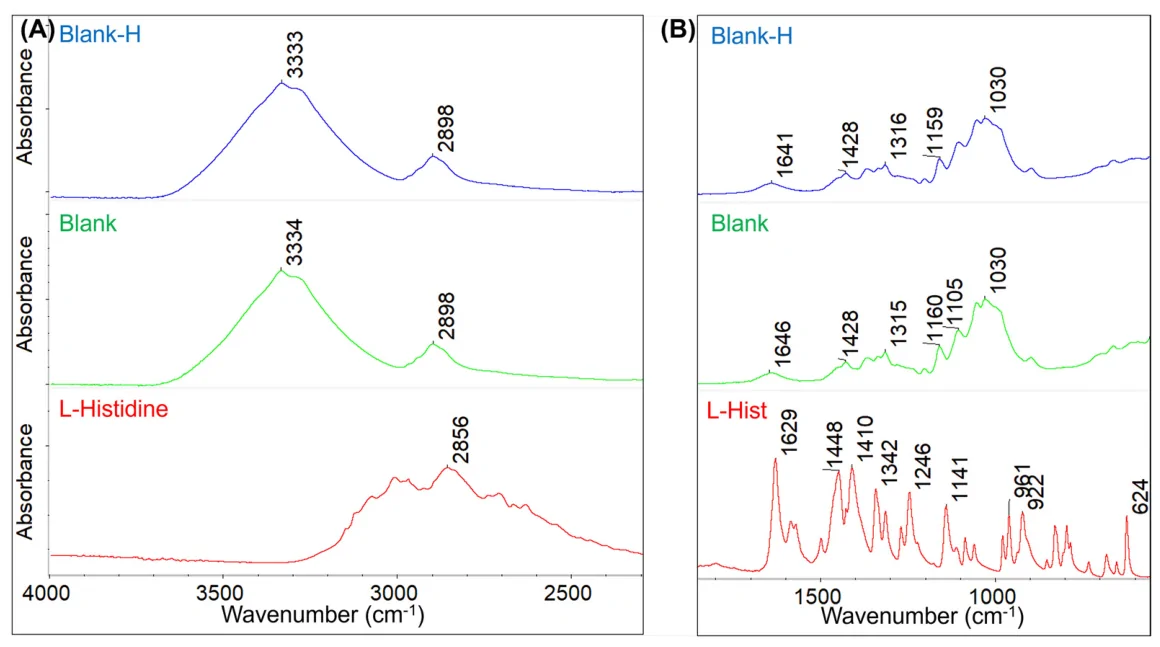
FTIR (ATR) spectra of Blank-H (blue spectrum, top), Blank (green, centre), and L-histidine (red, bottom). Blank-H was treated under reaction conditions (140 °C, 30 min). Spectral regions: (**A**) 4000–2600 cm^−1^, (**B**) 1800–550 cm^−1^.

The initial step is to determine whether histidine undergoes any chemical reaction with MFC; therefore, a comparison between FTIR spectra of Blank and Blank-H samples is made and reported in Figure 2 below, together with the pure histidine spectrum. The Blank sample was obtained by simply drying MFC under air for 3 days, while Blank-H was obtained under reaction and purification conditions. As clearly visible in Figure 2, Blank and Blank-H present superimposable spectra, and no signal belonging to histidine can be detected in the Blank-H sample. This means that no reaction occurs between MFC and histidine under the tested conditions, and the washing procedure is capable of removing the catalyst.

After confirming that no reaction occurs between histidine and MFC, it is possible to analyse the FTIR spectra of the reaction products to investigate whether the desired reaction has taken place, and to use the Blank-H spectrum as a reference for non-reactive MFC.

**Figure 3:**
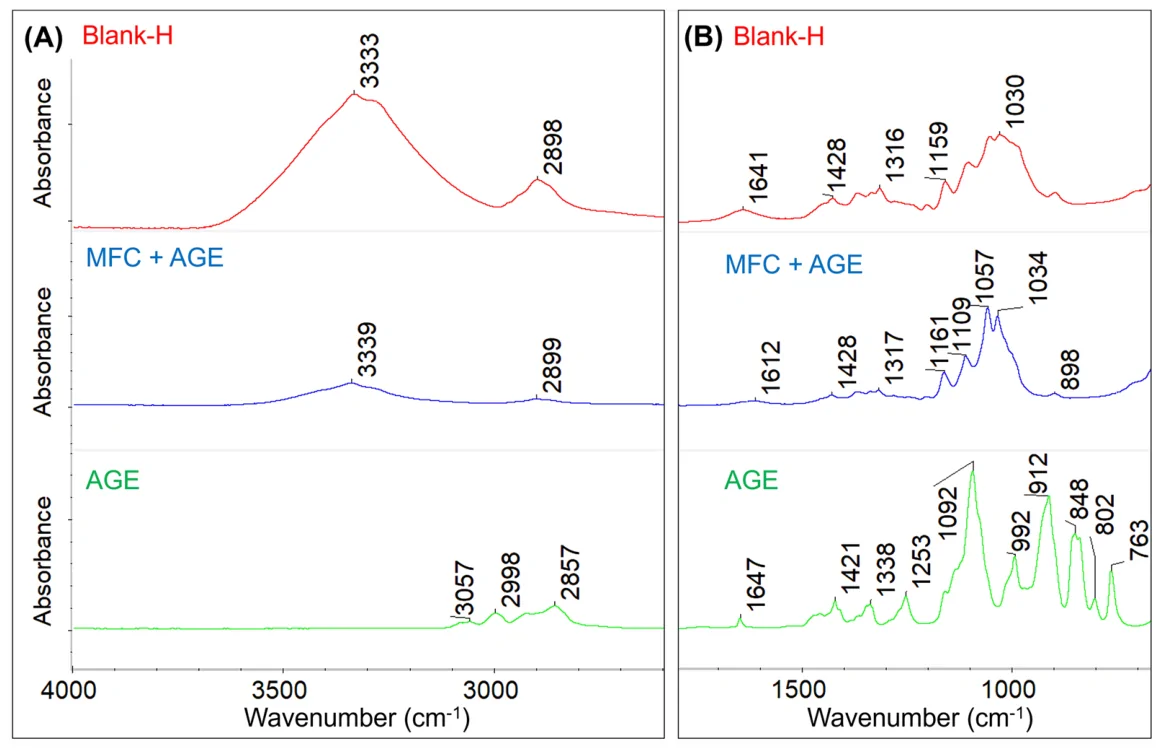
FTIR (ATR) spectra of Blank-H (top, red spectrum), MFC + AGE (P-AGE, centre, blue spectrum), and allyl glycidyl ether (AGE, bottom, green spectrum). Spectral regions: (**A**) 4000–2600 cm^−1^, (**B**) 1800–700 cm^−1^.

In Figure 3 below, the comparison between FTIR spectra of Blank-H (reference), P-AGE (MFC + AGE), and AGE is reported, with a zoom in the regions where the most important signals are recorded.

The only detectable differences between the FTIR spectra of Blank-H and P-AGE are the slight shifts of some signals, in particular the H-bonding band at around 3340 cm^−1^, the primary alcohol bending at 1317 cm^−1^, the stretching of the β(1→4) glycosidic linkage at 1160 cm^−1^, and the pyranose ring skeleton vibrations at 1110 cm^−1^. In fact, the main FTIR bands of AGE fall at the same wavenumbers as the main MFC signals. The only peak that is absent in the modified MFC spectrum is the epoxide signal of AGE at approximately 910 cm^−1^, indicating the opening of the oxirane ring. This evidence indicates that the epoxide is no longer present in the product; therefore, the possible reaction (see Figure 1) would produce an additional OH group (detected in the 3400 cm^−1^ region) and a new C-O bond (detected around 1300 cm^−1^), causing a shift of the signals in the product spectrum. Therefore, the detected shifts may indicate that the reaction has occurred; however, additional characterisation is performed to provide further confirmation of this reactivity.

**Figure 4:**
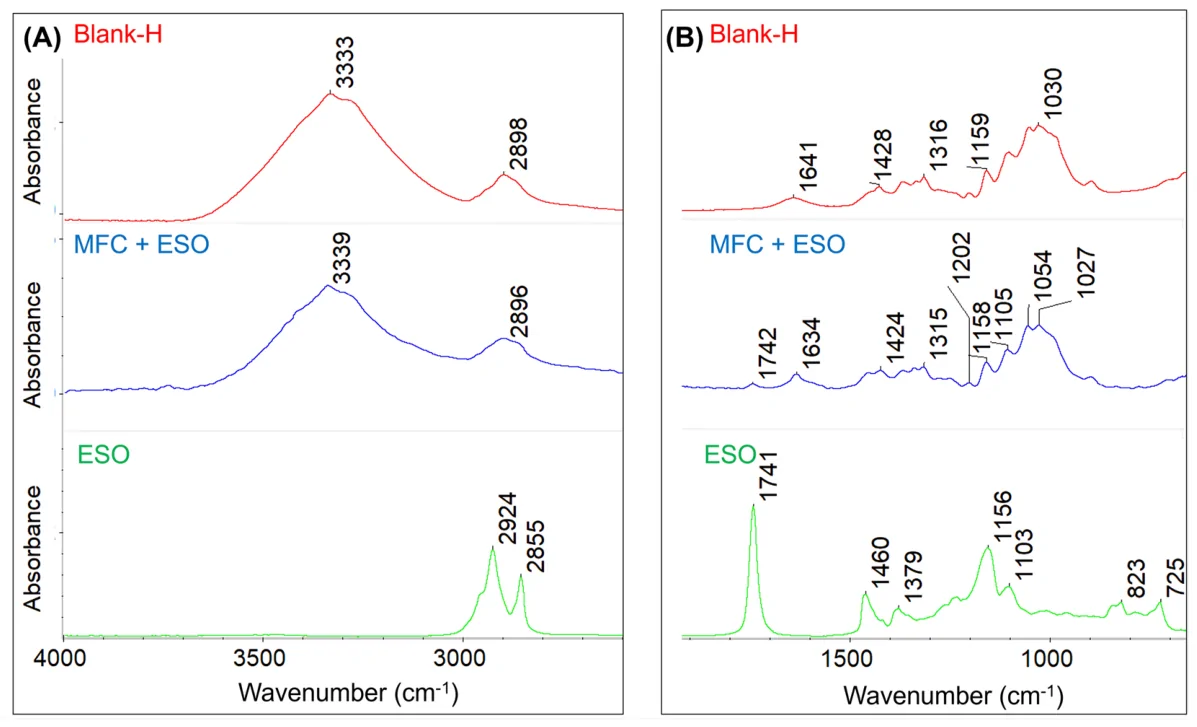
FTIR (ATR) spectra of Blank-H (top, red spectrum), MFC + ESO (P-ESO, centre, blue spectrum), and epoxidised soybean oil (ESO, bottom, green spectrum). Spectral regions: (**A**) 4000–2600 cm^−1^, (**B**) 1800–700 cm^−1^.

The same approach was used to characterise the product of the second model reaction, which tested the reactivity of MFC towards a bulky molecule (ESO). In Figure 4 below, the comparison between FTIR spectra of Blank-H (reference), P-ESO, and ESO is reported.

In this case, differences between MFC and the modified MFC are more visible: a new small band appears at approximately 1740 cm^−1^, attributed to the carbonyl stretching of the triglyceride of ESO [60,62], and most of the bands in the 1500–1110 cm^−1^ region are slightly shifted. Also, both the crystalline and the amorphous peaks are slightly affected after the reaction with ESO, indicating that this reaction may somehow affect the crystallinity of MFC. In fact, in the modified MFC, the additional functional groups can be considered a contribution to the amorphous region. Therefore, from the FTIR spectra, it can be concluded that the reaction between ESO and MFC has occurred.

In summary, FTIR characterisation provided evidence of MFC reactivity towards both AGE and ESO under identical reaction conditions. However, further characterisation of the products was performed to confirm that the MFC was successfully modified.

#### 3.1.2. TGA Analysis

TGA analyses were performed to further confirm the successful MFC modification and to investigate the degree of functionalisation.

**Figure 5:**
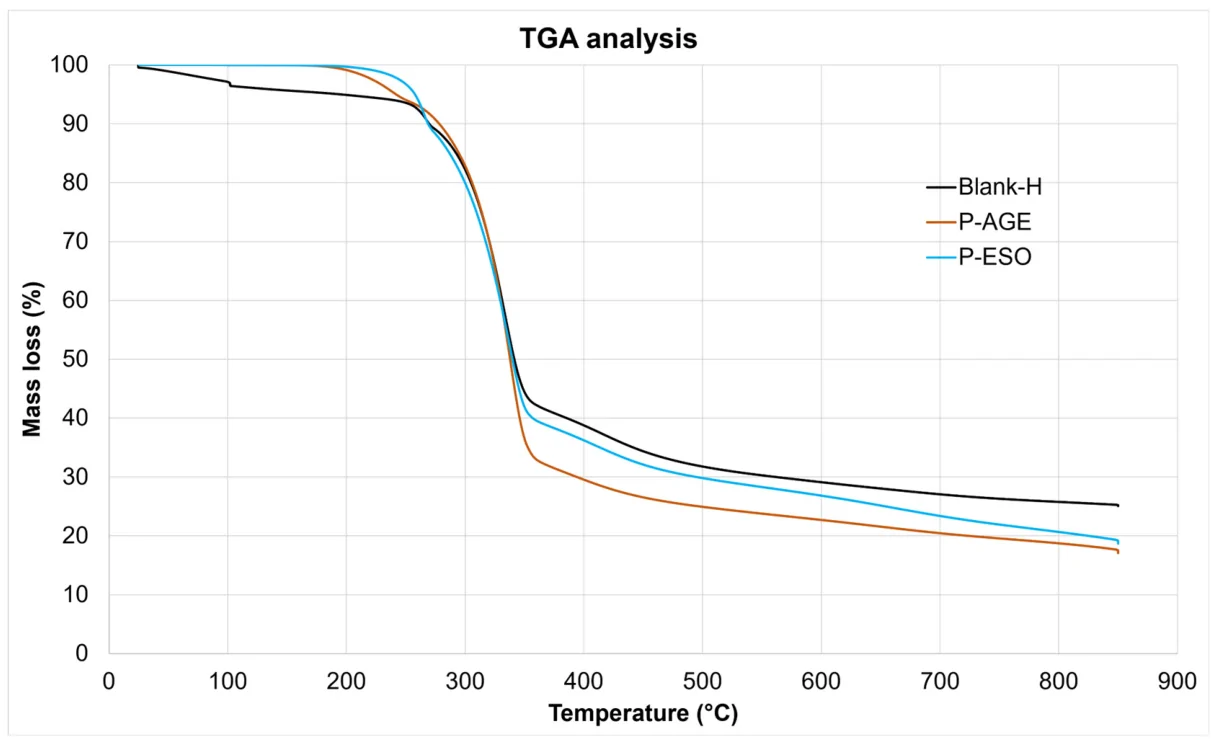
TGA curves of Blank-H (black curve), P-AGE (brown), and P-ESO (blue). Results from the last isothermal step under air are reported in Table 5 below.

In Figure 5 below, the thermogravimetric curves of Blank-H (black curve), Exilva P modified with AGE (P-AGE, brown curve), and Exilva P modified with ESO (P-ESO, blue curve) are shown, while in Table 5 the main mass loss steps are reported. For clarity of comparison, modified Exilva F samples are not reported, as their curves were superimposable on the reported ones.

The first noticeable evidence from Figure 5 and Table 5 is the mass loss before 150 °C, attributable to physisorbed (absorbed and adsorbed) water on the MFC surface. In fact, Blank-H shows around 4.3 wt% mass loss, in line with the literature data [69], while both P-AGE and P-ESO do not show any mass loss below 150 °C. This could be due to MFC surface modification: since both AGE and ESO are hydrophobic moieties, the modified MFC cannot adsorb water on its surface after the reaction.

The shapes of the curves are also different: P-AGE starts decomposing earlier than P-ESO, consistent with the different thermal stability of the two modifiers, but the onset point of the three curves is almost the same. This means that the main decomposition step in the pristine and modified MFCs occurs at nearly the same temperature, indicating that all MFC samples have similar thermal stability.

Another interesting piece of evidence is the final residue after burning the sample in air at 850 °C: unmodified MFC (Blank-H) presents more than 10 wt% of residue, while for both P-AGE and P-ESO the final residue is around 1 wt%. This means that the decomposition of the modified MFCs leads to the creation of different residues than for unmodified MFC, and these residues can be burned in air at elevated temperatures.

The main difference between the samples lies in the 150–850 °C region, where pristine MFC lost ca. 70 wt%, while P-AGE and P-ESO showed mass losses of 83 wt% and 81 wt%, respectively. Since this temperature range is where the decomposition of the additional functional group is expected, this could mean that the amount of modifier is around 13 wt% for P-AGE and ca. 11 wt% for P-ESO.

However, based on the reported data, it is not possible to clearly determine the degree of functionalisation of P-AGE and P-ESO. Nevertheless, all the evidence here reported suggests that the functionalisation reactions were successful.

#### 3.1.3. SEM Analysis

**Figure 6:**
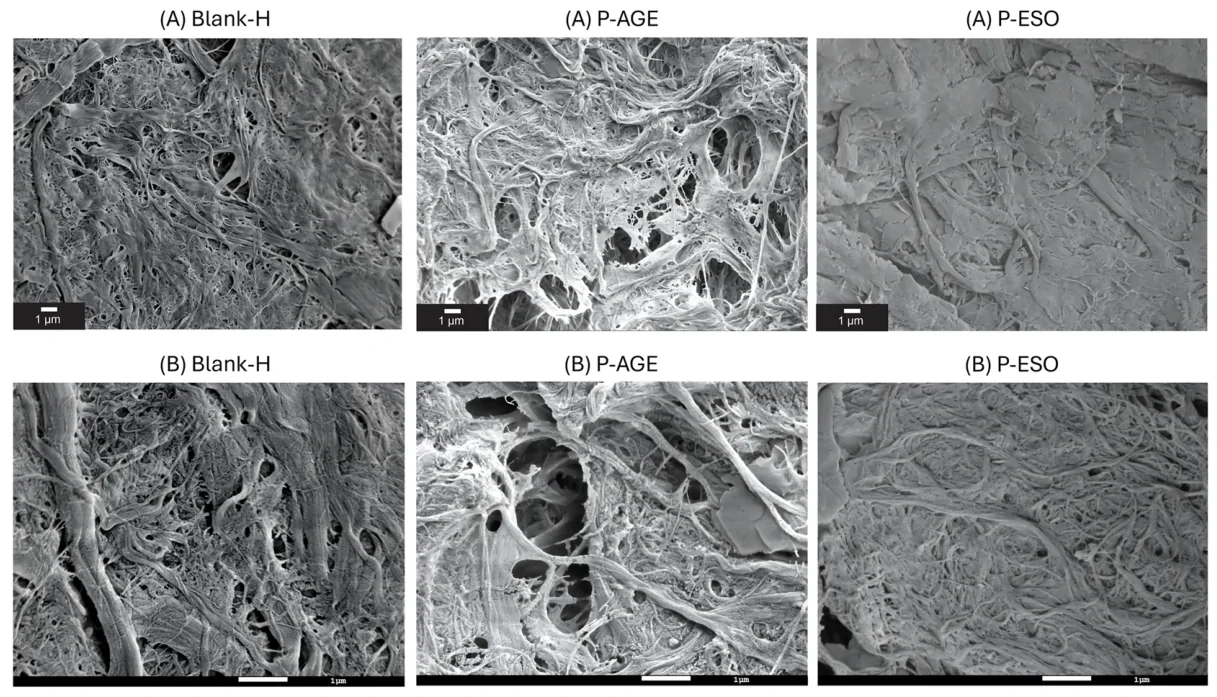
SEM images of Blank-H (**left**), P-AGE (**centre**), and P-ESO (**right**). Top pictures (**A**) were taken at lower magnifications, while bottom pictures (**B**) at higher magnifications (see the scale in the pictures).

SEM analysis was performed to assess whether and how the modification reactions affected the MFC surface. Micrographs are reported in Figure 6 below. Three samples were tested: one unmodified MFC (Blank-H), one MFC modified with AGE (P-AGE), and one MFC modified with ESO (P-ESO). Blank-H is used as a reference to fully confirm that the interaction with histidine has not caused any structural modification, even at the molecular level, since SEM images of unmodified MFC are already available in the literature [70,71,72].

The SEM images of Blank-H (Figure 6, left) show a typical MFC structure [70,71,72], with clean fibres randomly entangled, clearly distinguishable at any magnification. When the MFC is modified with AGE (Figure 6, centre), a small non-bulky probe molecule, the fibres are slightly coated but still recognisable. When the MFC is reacted with ESO (Figure 6, right), a bulky bio-based oil, the fibres are completely covered, and individual fibres can only be distinguished at very high magnification (Figure 6, bottom picture). This last result is fully consistent with the previous characterisation, in particular with the FTIR results, which showed a perturbation of the crystalline and amorphous peaks. In fact, the structure of P-ESO is clearly affected by the bulky modifier, which could be erroneously detected as an amorphous region by FTIR. In reality, the SEM images suggest that the crystallinity of the cellulose microfibrils is preserved. This result is in line with the literature data [62].

In summary, as clearly shown in the pictures in Figure 6, the modification of the MFC was successful under the chosen reaction conditions.

### 3.2. Rubber Compounds Filled with MFC (Or CB)

From the model studies previously reported, the reactivity of MFC is verified, the reaction conditions are established, and the role of histidine as a catalyst for MFC reaction towards epoxide groups is confirmed.

In this second part of the study, it will be investigated whether histidine can also enhance epoxy–MFC coupling in situ, to create innovative rubber compounds filled with 40 phr of MFC. Such an amount of filler, equal to approximately 25 wt%, is not expected to yield a high reinforcing effect; however, it is sufficient to appreciate the influence of MFC on compound properties and to compare them with CB-filled compounds [24]. Moreover, it seems that this is the highest amount of MFC reported as a filler in rubber compounds [5,6,24,28,36,73,74]. In fact, MFC is typically used in combination with other fillers (mainly silica and CB) in small amounts (2–15 phr), since the incorporation of more than 20 phr leads to agglomeration and flocculation issues, owing to incompatibility with the rubber matrix and the other fillers [26,75,76,77,78]. This new approach, which uses histidine as a catalyst in an ENR matrix, is expected to overcome this limitation. This is investigated in the following part.

Both grades of MFC (Exilva F and P) were used, this time in paste (-V) form (10 *w*/*w*% in water). The selected rubber matrix is epoxidised natural rubber (ENR) with an epoxidation level of 50 mol%. The role of histidine as a catalyst for the reaction is also investigated by adding the same amount as used in the model reaction (10 *w*/*w*%). Also, the mixing temperature is maintained at the same level as in the model reaction (140 °C) to ensure proper evaporation of water. Complete water removal is verified by TGA analysis of the final MFC-filled compounds, which show no weight loss below 200 °C. These results exclude the presence of “pockets” inside the ENR-based compounds, possibly formed due to the fast water evaporation during mixing. In fact, if some remaining steam were trapped inside the compound, TGA analysis would reveal a certain weight loss below 200 °C due to steam release.

As previously mentioned, the idea is to verify whether the reactivity of the MFC towards epoxide groups is sufficient to form a crosslinked network between the filler and the polymer. The creation of this network will affect the in-rubber properties of the MFC-filled compounds, possibly leading to a decreased Payne effect and hysteresis. The influence of this network on the compound’s processability will also be investigated in terms of S′min, a parameter related to compound viscosity.

As discussed above, the properties of these MFC-filled rubber compounds are compared to the same compounds where MFC is fully substituted (40 phr) by CB N330, a commercial grade of reinforcing filler, mixed under the same conditions. CB is considered a non-reactive filler, and blank reaction with histidine showed no reactivity between the two species. Therefore, one additional CB-filled compound is produced, with the addition of histidine in the same amount (4 phr) as for MFC-filled compounds, to investigate the influence of histidine on in-rubber properties in the absence of a reactive filler.

Formulations of the rubber compounds are reported in Table 1, while mixing procedures are reported in Table 2 and Table 3 above.

Cure behaviour, dynamic-mechanical and tensile properties of the rubber compounds are investigated in the following. As described above, two CB-filled compounds (without histidine addition) are reported as references, one mixed at 80 °C (CB N330@80C), the other at 140 °C (CB N330@140C), to investigate the effect of first-stage mixing temperature on the compounds’ properties.

#### 3.2.1. Cure Behaviour

**Figure 7:**
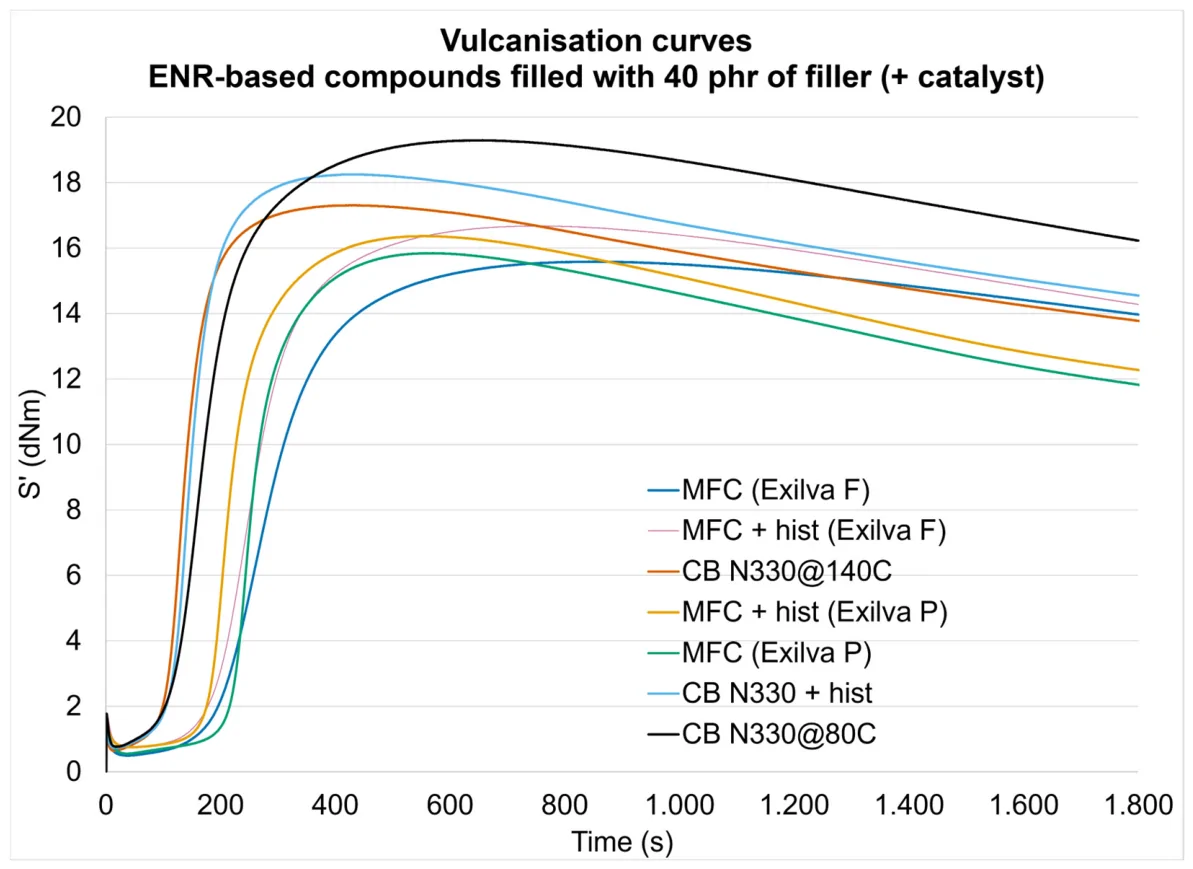
Vulcanisation curves of the compounds reported in Table 1. ENR-based compounds filled with 40 phr of CB N330@140C (vermilion curve), CB N330@80C (black), CB N330 + 4 phr of histidine (sky blue), MFC (Exilva F, blue), MFC (Exilva P, bluish green), MFC (Exilva F) + 4 phr histidine (reddish purple), and MFC (Exilva P) + 4 phr of histidine (orange). Vulcanisation made at T = 160 °C, frequency of 1.667 Hz and 6.98% strain.

In Figure 7 below, the vulcanisation curves of the compounds listed in Table 1 are reported, while Table 6 gives an overview of the main results of this test.

As shown in Figure 7 and Table 6, the two MFC grades (without histidine addition) behave in the same way in terms of cure properties: the corresponding compounds show very similar S′max and S′min, scorch times (ts2), and cure times (t90). Only the vulcanisation rate values (difference between t90 and ts2) obtained using Exilva F are slightly higher than for Exilva P. Moreover, the addition of histidine has the same effect on both MFC-filled compounds: it increases the vulcanisation rate (decreased scorch time and t90) and enhances the final torque (S′max). Therefore, histidine appears to act as a catalyst when added in situ to ENR-based MFC-filled rubber compounds, at least in boosting vulcanisation. Furthermore, the effect on the compounds’ processability appears to be negligible, since the S′min values are similar, indicating that their viscosity is not significantly changed.

**Figure 8:**
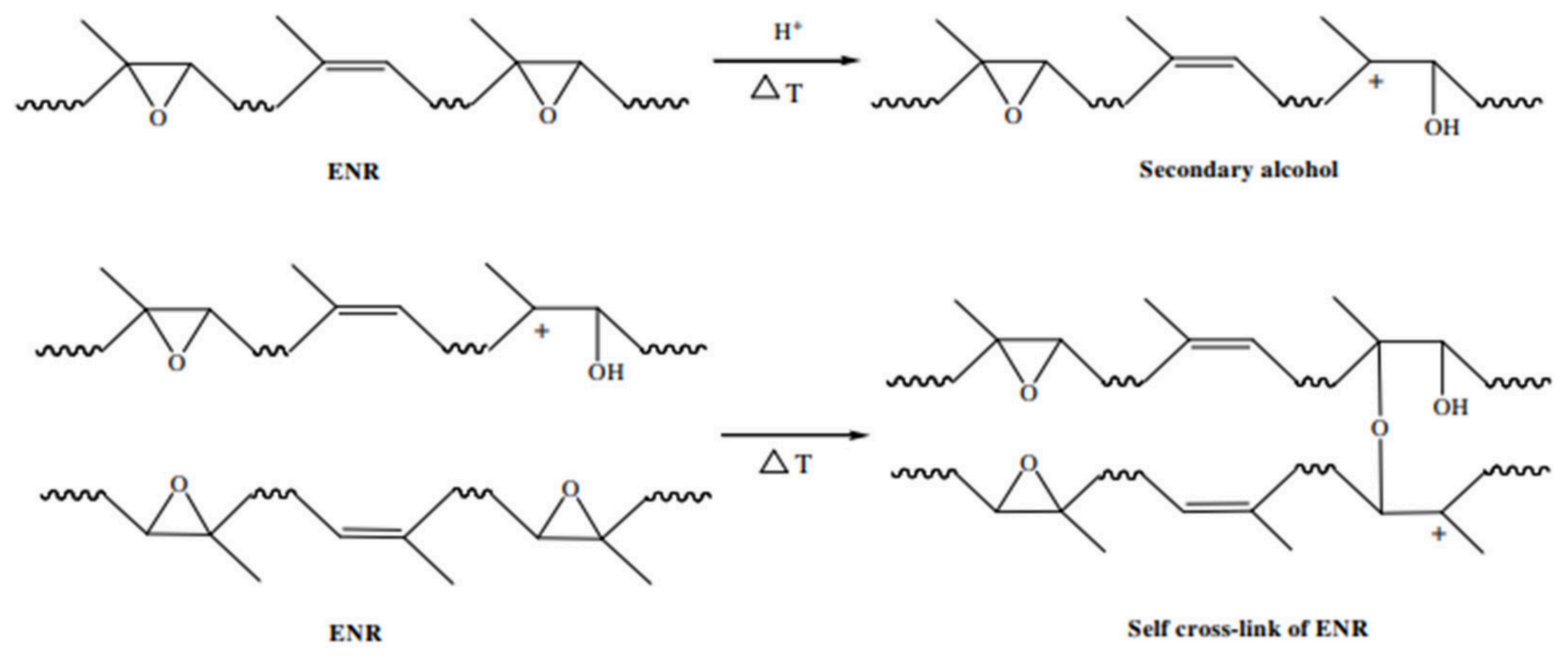
General mechanism for epoxide self-crosslink (condensation) [79,82].

By contrast, when histidine is added to CB N330, the catalytic effect is no longer observed: the scorch time is unaffected, t90 slightly increases, and the slope of the cure curve is identical to that of the CB-filled compound without histidine. One noticeable effect is the increase in S′max. This could be explained by the self-condensation (self-crosslinking) of the epoxide groups of ENR, promoted by histidine, which may facilitate epoxide ring opening and increase the probability of intramolecular reactions between polymer chains, as reported in Figure 8 below [79]. This effect was already observed in the literature when curing ENR in the presence of imidazole-containing molecules [80], and also in unfilled ENR-based compounds [81].

A final comment regards the effect of first-stage mixing temperature on CB N330-filled compounds: the vulcanisation behaviour of the CB-filled compound mixed at 140 °C is similar to that of MFC-filled compounds mixed at the same temperature, while the CB-filled compound mixed at 80 °C shows higher S′max and S′min. This fact was expected, since the lower mixing temperature affects the viscosity of the ENR (as demonstrated by an increased S′min), leading to higher shear force inside the mixer, which facilitates the incorporation of CB aggregates inside the matrix; therefore, the compound develops a higher maximum torque (and possibly a higher crosslink density) during vulcanisation.

Surprisingly, the cure characterisation of MFC-filled compounds yielded results similar to those of CB-filled compounds (mixed at the same temperature): similar S′min, slightly lower S′max, but increased scorch time and t90, indicating safer processability of MFC-filled compounds.

#### 3.2.2. Strain Sweep Results

**Figure 9:**
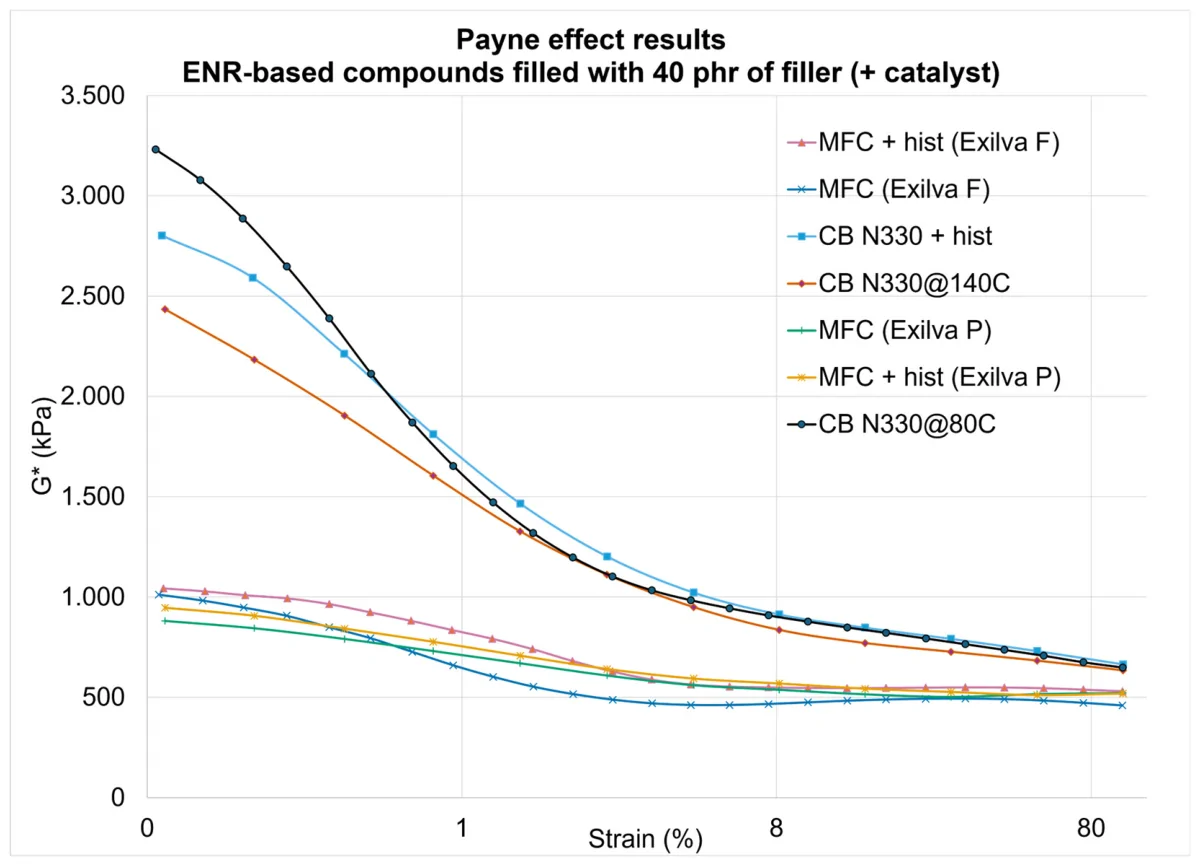
Cured Payne effect results of the compounds reported in Table 1. ENR-based compounds filled with 40 phr of CB N330@140C (vermilion curve), CB N330@80C (black), CB N330 + 4 phr of histidine (sky blue), MFC (Exilva F, blue), MFC (Exilva P, bluish green), MFC (Exilva F) + 4 phr histidine (reddish purple), and MFC (Exilva P) + 4 phr of histidine (orange). Vulcanisation made at T = 160 °C, t90. High-to-low sweep (1.67 Hz, 60 °C).

Figure 9 below reports the complex modulus (G*) dependence on strain (%) for the compounds listed in Table 1, while Table 7 shows the main results obtained from the test, including storage modulus (G′), loss modulus (G′′), and tan delta minimum and maximum values.

As expected, CB-filled compounds display the highest levels of reinforcement, with higher complex moduli than MFC-filled compounds at all strains. In fact, CB N330 is considered a reinforcing filler, commonly used in tyre compounds. When added to rubber compounds, it creates a strong filler–filler network that is broken as strain increases, causing a fast decrease in complex moduli (Payne effect). The stronger the filler–filler network, the higher the strain needed to break it. This is why the two CB-filled compounds behave differently in terms of the Payne effect. As reported above, a lower mixing temperature during the first mixing stage leads to the creation of a stronger filler–filler network, which is recorded as an increased Payne effect. However, the filler–polymer network is not affected, as demonstrated by the same G*min (at 100% strain).

The addition of histidine to CB increases G* at all strains, as well as the Payne effect (ΔG*). Moreover, the onset of the Payne effect is shifted to higher strains, indicating a stronger filler–filler network in the presence of histidine. As previously hypothesised, this could be due to self-condensation of the epoxide groups of ENR (see Figure 8), which creates intramolecular crosslinks between polymer chains, increasing the complex modulus of the compound.

Regarding MFC-filled compounds, both grades show similar behaviour, with G*max (at 0.1% strain) values of around 900–1000 kPa and G*min (at 100% strain) of 450–500 kPa, and consequently a much lower Payne effect (ΔG*) than CB-filled compounds, but also a lower reinforcing effect. The addition of histidine to MFC-filled compounds slightly increases the complex moduli at all strains, while keeping the Payne effect at similar values to those of pure MFC. Based on these data, it is not possible to discriminate between the catalytic effect of histidine on epoxide self-crosslinking and its possible promotion of the reaction between ENR and MFC. However, epoxide self-condensation alone would have caused a large increase in the Payne effect, as observed for CB-filled compounds, especially at such a high MFC loading [24]. Therefore, histidine appears to promote MFC dispersion, possibly by catalysing the ENR–MFC reaction. It remains unclear whether the reaction occurs preferentially during mixing or vulcanisation. Further studies are needed to elucidate this aspect.

Considering the loss moduli and tan delta data reported in Table 6, it is clear that MFC-filled compounds exhibit a different viscoelastic behaviour from CB-filled compounds: G′ and G″ values are considerably lower, as are tan delta values. This could be a positive outcome, as it may indicate that MFC-filled compounds have lower hysteresis than CB-filled compounds, and could therefore dissipate energy more efficiently when subjected to external forces. In fact, tan delta values at 60 °C are commonly correlated with compound hysteresis and, consequently, rolling resistance. This could be a further confirmation of the role of histidine: in the case of ENR epoxide self-condensation, tan delta values would be expected to increase, as recorded for the CB-filled compound.

To better investigate the reinforcing behaviour of the compounds and the role of histidine, tensile and hardness tests were performed.

#### 3.2.3. Tensile and Hardness Tests

**Figure 10:**
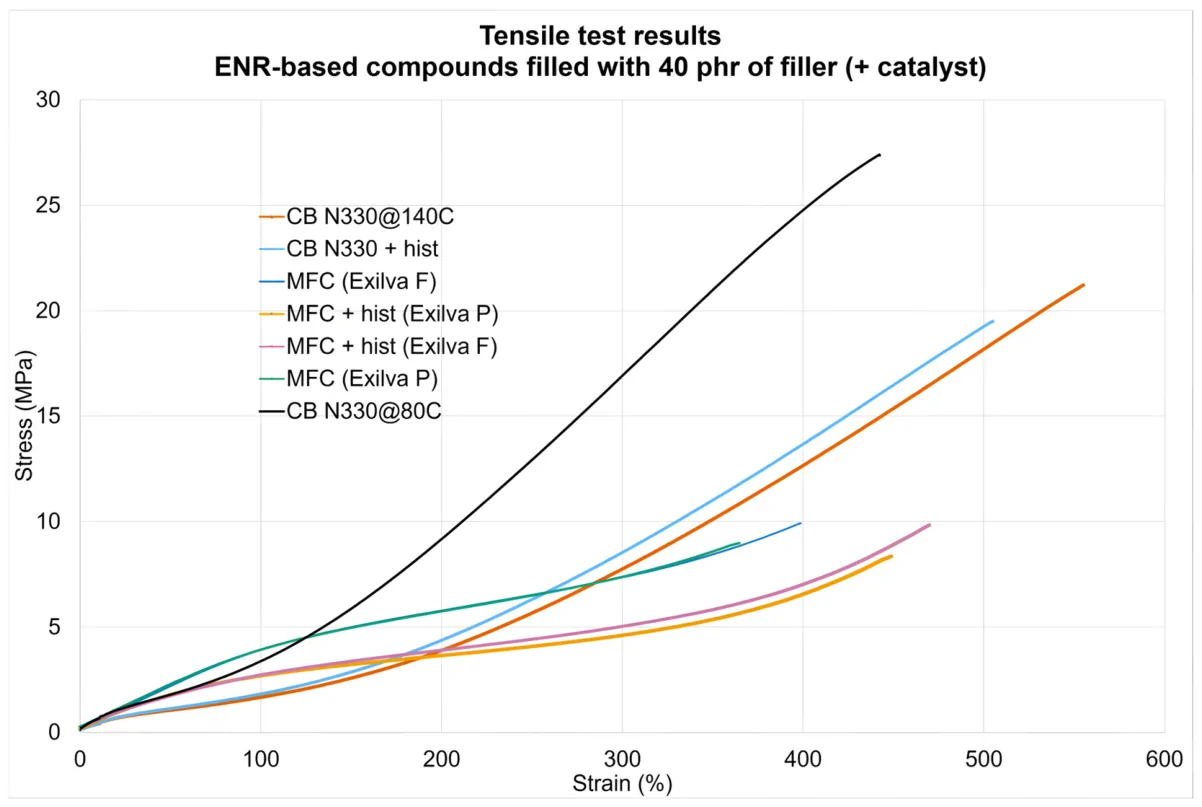
Tensile test results of the compounds reported in Table 1. ENR-based compounds filled with 40 phr of CB N330@140C (vermilion curve), CB N330@80C (black), CB N330 + 4 phr of histidine (sky blue), MFC (Exilva F, blue), MFC (Exilva P, bluish green), MFC (Exilva F) + 4 phr histidine (reddish purple), and MFC (Exilva P) + 4 phr of histidine (orange). Vulcanisation made at T = 160 °C, t90.

Figure 10 shows the stress–strain curves obtained for the compounds listed in Table 1, while in Table 8 the main results of elongation at break, modulus at break, modulus at 100% and 300% strain, stiffness (M300/M100), and hardness (Shore A) values are reported with their standard deviations.

As clearly visible from Figure 10, MFC-filled compounds have different tensile properties compared to CB-filled compounds. Additionally, the data in Table 8 make it clear that the differences between the curves are not within the samples’ standard deviation, which is always relatively low.

CB-filled compounds exhibit a typical stress–strain behaviour of such elastomeric compounds, with a linear increase of moduli after 150% strain. ENR-based compound filled with 40 phr of CB N330 mixed at 140 °C (vermilion curve) reaches the highest values of elongation at break (560%), while the CB N330 compound mixed at 80 °C (black curve) reaches the highest modulus at break (27 MPa). These results are in line with the literature data on similar compounds [83]. The difference between these two compounds highlights once again the impact of first-stage mixing temperature on the compound’s properties: lower mixing temperature increases the S′max of the compound (see Figure 7 above), which is correlated to its crosslink density. This provokes a higher Payne effect (see Figure 9 above), and therefore higher filler–filler interactions. Higher crosslink density and a higher filler–filler network led to a higher modulus at break and hardness but a lower elongation at break, as reported in Figure 10 and Table 8.

The addition of histidine to CB N330 (sky blue curve) causes a reduction of around 10% in both elongation (ca. 500%) and modulus at break (19.3 MPa). This significant decrease in tensile properties seems to confirm the hypothesis that histidine promotes self-condensation of epoxide groups in ENR, leading to intramolecular crosslinks which can slightly contribute to increasing the maximum torque during vulcanisation [81], but have a negative impact on the polymer–filler network (increasing the Payne effect), and therefore on tensile properties. In fact, these crosslink sites are shorter than most of the sulphur bridges (apart from monosulphidic crosslinks) and therefore less elastically active [81,83,84].

This theory is also supported by the hardness values. In fact, when histidine is added to the CB-filled compound, the hardness increases from 57 to 63. This increment is outside the standard deviation of the samples, indicating that the compound becomes more rigid. However, the stiffness (M300/M100) derived from the tensile test is the same for both compounds filled with CB and CB + histidine (ca. 4.5); therefore, the increased hardness can be due to the creation of intramolecular crosslinks between polymeric chains after ENR epoxide self-crosslinking.

Regarding MFC-filled compounds, the two cellulose grades (Exilva P and F) have similar tensile behaviour, with Exilva F achieving slightly higher elongation (ca. 400%) and modulus at break (10.1 MPa) with respect to Exilva P. The moduli at 100% strain for both MFC-filled compounds are the highest, then plateau until 300% strain, after which they increase again. This behaviour is typical for MFC-filled compounds, and the plateau is generally attributed to reduced interfacial adhesion and limited stress transfer at the filler–matrix interface under deformation, particularly in comparison to conventional CB-filled compounds [24,36]. For this reason, the stiffness (M300/M100) of the MFC-filled compounds is the lowest: both moduli at 100% and 300% strain have similar values; therefore, the ratio is smaller than for CB-filled compounds, where the moduli were increasing together with the strain.

The addition of histidine to MFC significantly influences tensile properties, shifting the curves to higher elongations while reaching nearly the same stress at break. In fact, both Exilva P and F show an approximately 15% increase in elongation at break upon histidine addition, reaching 440% and 470%, respectively. Stress at break remains at similar values: around 9.5–10 MPa for Exilva F, and approximately 8–9.5 MPa for Exilva P. These results suggest that histidine improves MFC dispersion within the ENR matrix by forming a more effective polymer–filler network, possibly enhancing epoxide ring opening and subsequent reaction with the OH groups of MFC. This network improves the tensile properties of the corresponding compounds, increasing interfacial adhesion and stress transfer, as evidenced by the higher elongation at break. The comparable stress values at break indicate that the crack-formation mechanism is similar across all cases. This implies that the newly formed MFC–ENR network is probably too rigid and constrained. Therefore, a “spacer” in between MFC and ENR could be beneficial. Such a spacer could be a coupling agent, or an oil (e.g., ESO), or a (functionalised) liquid polymer, which could be bonded in between MFC and ENR by exploiting the here reported reactivity.

Regarding the hardness (Shore A), all MFC-filled compounds obtained the same values (62–64), and the differences are within the standard deviation of the samples. This means that the addition of histidine to MFC does not increase the hardness of the compound, which is further proof that histidine does not catalyse epoxide self-condensation in ENR, but the ENR–MFC reaction.

In light of these considerations, the different behaviour of CB and MFC in the presence of histidine can be explained as follows: for unreactive fillers (such as CB), histidine catalyses epoxy self-condensation, shifting the tensile curve to the left (lower elongation and stress at break) and increasing compound hardness, while for reactive fillers (such as MFC), it can enhance polymer–filler interactions, shifting the tensile curves to the right (higher elongation and stress at break) and not influencing the hardness of the compound.

To better elucidate this aspect, and more generally the dispersion of MFC within ENR-based compounds, SEM analyses are performed.

#### 3.2.4. SEM Analysis

SEM images are collected on two different rubber compounds: one MFC-filled compound and one CB-filled compound, both mixed at 140 °C and in the presence of histidine. In fact, the idea is to visualise the different dispersion of the fillers inside the ENR matrix and also possible defects, cracks or filler agglomeration due to ENR epoxide self-condensation. Since the two MFC grades (Exilva P-V and F-V) gave similar results from the previous characterisations, just one sample was selected for imaging (Exilva P). Specimens were taken from vulcanised tensile sheets and polished using the procedure described above (Section 2.3.3).

**Figure 11:**
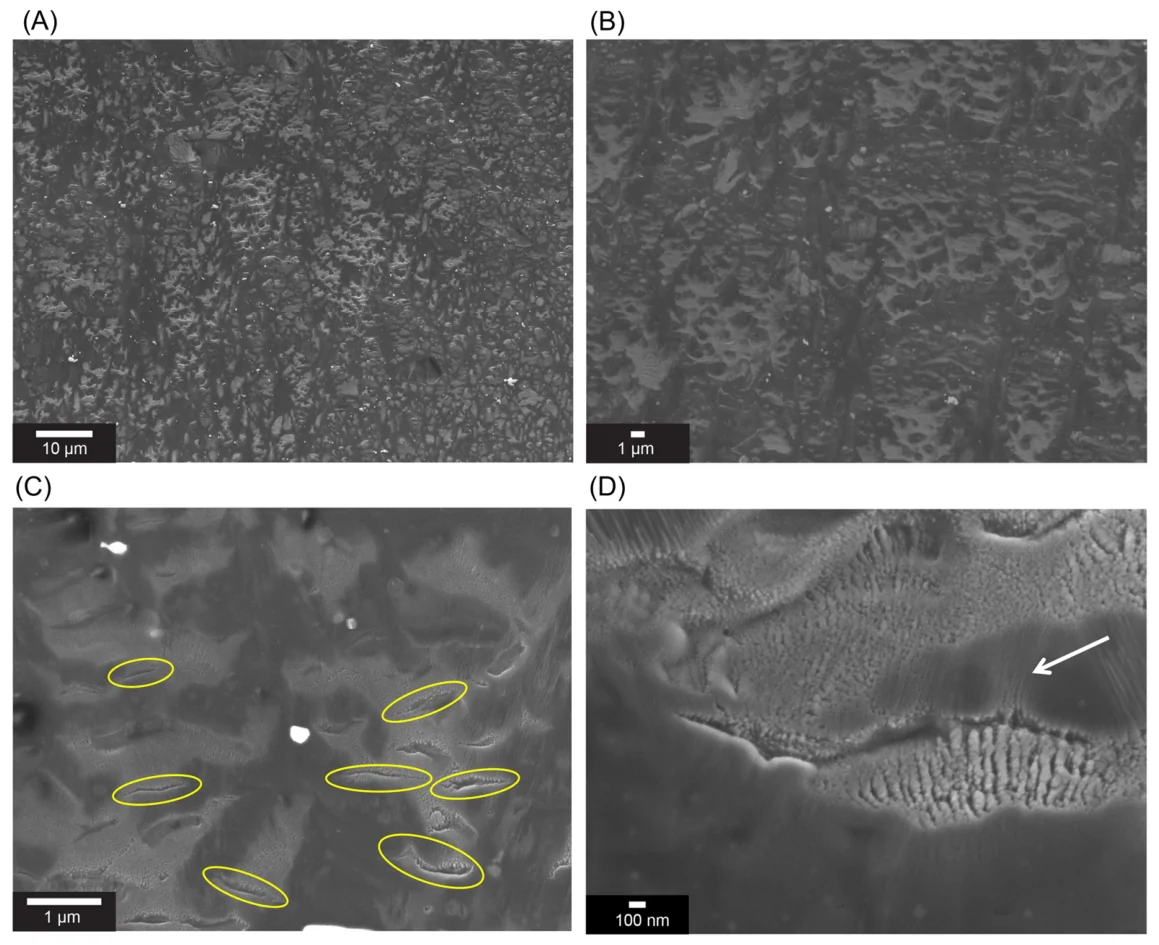
SEM images of MFC-filled compound (Exilva P) with histidine, taken at different magnifications, increasing from (**A**) (top left) to (**D**) (bottom right). The yellow circles in (**C**) highlight some of the MFC fibrils. The arrow marks curtaining artefacts caused by the ion beam polishing process during sample preparation (detectable in all images at high magnification).

Figure 11 reports the SEM images of the MFC-filled compound (Exilva P) with the addition of histidine. Magnification increases from top left to bottom right.

As clearly visible in Figure 11, the cellulose microfibrils are homogeneously dispersed within the ENR matrix. In fact, especially in the two images at the top, taken at lower magnification, no large cellulose clusters are present in the compound. On the contrary, the microfibrils appear to be well distributed within the matrix, as they are easily identified at higher magnification (Figure 11C, yellow circles). Moreover, they appear to be completely surrounded by the matrix, since neither voids nor cracks can be detected at the nanometric scale.

The effect of ENR epoxide self-condensation is to create short crosslinks between the polymeric chains [79,81,82] (see Figure 8). These crosslinks could lead to crack formation inside the compound, especially if they involve neighbouring chains (not to be excluded since the epoxidation level is 50 mol%), which is not the case here. On the contrary, neither defects in the compound nor microfibril clustering are observed, which is remarkable given such a high MFC loading. Therefore, it can be concluded that the predominant role of histidine in MFC-filled compounds is to promote MFC dispersion, possibly by catalysing the reaction between MFC and ENR. In fact, the homogeneous distribution of MFC can be explained by the reaction with ENR epoxides: each epoxide is a reactive site that accepts just one MFC fibre. If the MFC–ENR interaction did not involve a chemical bond (e.g., H-bonding), then MFC would create clusters since the material is already subjected to a strong intermolecular H-bonding network, which would drive MFC fibres towards clustering.

**Figure 12:**
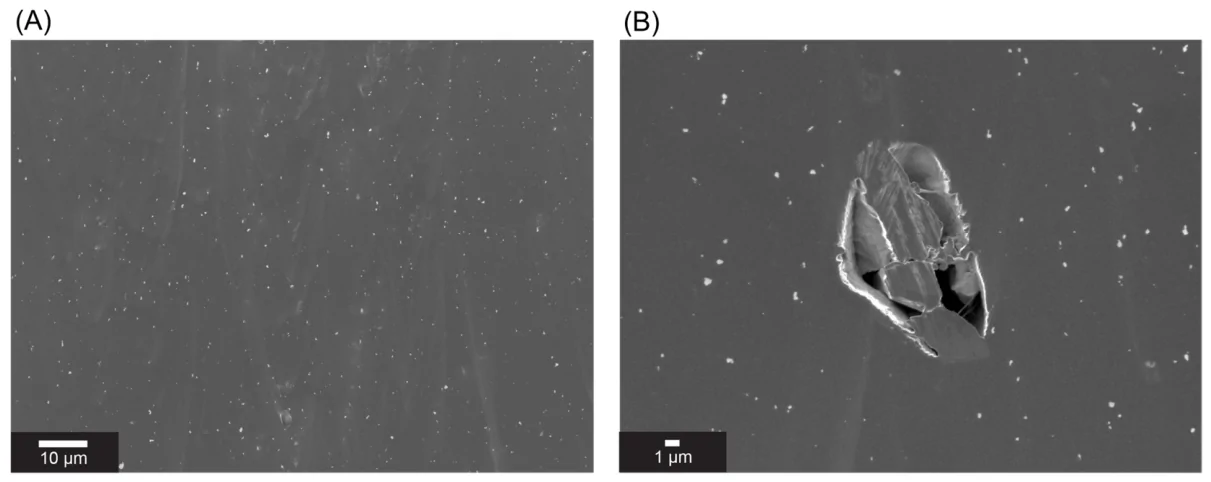
SEM images of CB-filled compound (CB N330) with histidine, taken at different magnifications (increasing from (**A**) and (**B**)).

Based on these hypotheses, it is now possible to analyse the SEM images of the CB-filled compound with histidine (Figure 12 below), taken at different magnifications (higher from left to right, see the scale in the pictures).

SEM images reported above confirm that the dispersion of CB is optimal, since neither clusters nor agglomerates can be distinguished. This was expected for such a low amount of CB (40 phr). However, the higher-magnification image (Figure 12B) highlights a crack in the compound that is not visible in the MFC-filled compound. This defect can be due to ENR epoxide self-condensation, which forms short bonds between polymeric chains, potentially leading to cracking during compound vulcanisation.

It remains unclear whether the self-crosslinking of the epoxide in NR (see Figure 8) occurs during mixing or vulcanisation. However, considering that this effect is not observed in MFC-filled compounds, it can be assumed that it preferentially takes place during vulcanisation.

Further characterisations are needed to better clarify this aspect.

## 4. Conclusions

This research proposes a novel approach to producing MFC-filled rubber compounds that exploits the surface chemistry of cellulose to form a stable polymer–filler network using epoxidised natural rubber (ENR).

In the first part of this study, the reactivity between MFC and epoxide-containing molecules is verified through model reactions. A probe molecule (allyl glycidyl ether, AGE) is first used as an MFC modifier to establish reaction conditions suitable for transfer into an internal mixer and to assess the catalytic effect of histidine, a natural amino acid. Once the reaction conditions are defined, MFC modification is performed using epoxidised soybean oil (ESO), a bulky molecule having long apolar chains. The success of the synthesis is confirmed through FTIR, TGA, and SEM analysis. The identified reaction conditions are environmentally friendly, since the only solvent used is water (apart from a small amount of ethanol), reaction times are short (30 min), and the temperature is relatively low (140 °C), and they are directly transferable to an internal mixer.

In the second part, the reactivity of MFC towards ENR is tested directly in a small internal mixer (Brabender) under the same conditions. MFC-filled compounds are produced in a two-step mixing process, and their properties are compared to those of compounds where the same amount of MFC (40 phr) is replaced by CB N330, a common reinforcing filler for tyre compounds. The cure characterisation reveals that MFC-filled compounds exhibit safer processability than CB-filled compounds, with increased scorch time and cure time (t90), while minimum and maximum torques remain at comparable values, indicating similar compound viscosity and crosslink density. The effect of histidine varies with the filler: In the presence of MFC, it increases the vulcanisation rate while preserving safe processability and enhancing the final torque (S′max); in the presence of CB, it affects only the maximum torque. This suggests that histidine catalyses epoxide ring opening, promoting ENR–MFC reactivity in the presence of a reactive filler, and favouring ENR self-condensation in the presence of a non-reactive one.

This hypothesis is supported by the strain-sweep data. As expected, CB yields the highest reinforcement, as indicated by the highest G* values at all strains, but the addition of histidine causes an increase in ΔG*, consistent with ENR self-condensation. In contrast, for MFC-filled compounds, the Payne effect decreases upon histidine addition, as well as compound hysteresis, indicating improved polymer–filler interaction and enhanced filler dispersion, attributable to ENR–MFC reactivity rather than epoxide self-condensation.

Further evidence comes from tensile characterisation. In line with expectations, CB-filled compounds show superior tensile behaviour, with higher stress transfer, moduli, and elongations at break with respect to MFC-filled compounds. When histidine is added to CB-filled compounds, it slightly deteriorates tensile properties, reducing both modulus and elongation at break, and increasing the hardness of the compound, consistent with the formation of elastically inactive intermolecular crosslinks via self-condensation of natural rubber epoxides. Conversely, when added to MFC-filled compounds, histidine increases elongation at break while preserving the modulus and the hardness, consistent with enhanced ENR–MFC interaction and improved filler dispersion.

Finally, SEM analysis confirms the homogeneous distribution of microfibrils within the ENR matrix, with no evidence of cluster formation or flocculation, further validating this approach. By contrast, SEM images of the CB-filled compound with histidine revealed cracks resulting from epoxide self-crosslinking.

In summary, a novel strategy to produce MFC-filled ENR-based elastomeric compounds is presented that exploits cellulose surface chemistry and the epoxide functionality of ENR to create a stable polymer–filler network, with histidine serving as a sustainable catalyst. This strategy enables the in situ incorporation of 40 phr of MFC into ENR-based compounds without any additional mixing step. To the best of our knowledge, this represents the first successful incorporation of such a high MFC content into a rubber compound, confirming the effectiveness of this approach. Although the reinforcing effect of MFC is slightly inferior to that of CB, MFC-filled compounds offer several advantages: they are more sustainable, as they contain no fossil-based filler, biocompatible, and lightweight, owing to the lower density of MFC (1.5 g/cm^3^) compared to CB (1.8 g/cm^3^).

This proof-of-concept study demonstrates the ready reactivity and compatibility of MFC in ENR compounds, laying the groundwork for a new class of bio-based composites, and possibly paving the way to a new generation of sustainable tyre materials.

## Figures and Tables

**Table 1:** Formulation of the rubber compounds.

	CB N330	CB N330 + Hist	MFC + Hist	MFC (Blank)
Raw material	phr	phr	phr	phr
ENR 50	100	100	100	100
Exilva F-V or P-V	/	/	40	40
CB N330	40	40	/	/
Histidine	/	4	4	/
ZnO	4	4	4	4
Stearic acid	2	2	2	2
TBBS	1.8	1.8	1.8	1.8
6PPD	2	2	2	2
Sulphur	1.5	1.5	1.5	1.5

**Table 2:** Mixing procedure for the production of MFC-filled (Exilva P-V and Exilva F-V) rubber compounds.

Time (min)	Action
Step 1. Initial temperature: 140 °C, rotor speed: 50 rpm/variable	
0:00–1:00	Addition rubber, mastication
1:00–2:00	Addition ¼ filler (+¼ catalyst)
2:00–3:30	Mixing
3:30–4:30	Addition ¼ filler (+¼ catalyst)
4:30–6:00	Mixing
6:00–7:00	Addition ¼ filler (+¼ catalyst)
7:00–8:30	Mixing
8:30–9:30	Addition ¼ filler (+¼ catalyst)
9:30–12:00	Mixing, discharge at 140 °C
Step 2. Initial temperature: 50 °C, rotor speed: 50 rpm	
0:00–1:00	Load masterbatch, mastication
1:00–1:20	Addition ZnO, stearic acid, TBBS
1:20–3:00	Mixing
3:00–3:20	Addition sulphur, 6PPD
3:20–5:00	Mixing, discharge at 50 °C

**Table 3:** Mixing procedure for the production of CB-filled rubber compounds.

Time (min)	Action
Step 1. Initial temperature: 80 °C (or 140 °C), rotor speed: 50 rpm	
0:00–1:00	Addition rubber, mastication
1:00–1:20	Addition ½ filler (+½ catalyst)
1:20–2:00	Mixing
2:00–2:20	Addition ½ filler (+½ catalyst)
2:20–5:00	Mixing, discharge at 80 °C (or 140 °C)
Step 2. Initial temperature: 50 °C, rotor speed: 50 rpm	
0:00–1:00	Load masterbatch, mastication
1:00–1:20	Addition ZnO, stearic acid, TBBS
1:20–3:00	Mixing
3:00–3:20	Addition sulphur, 6PPD
3:20–5:00	Mixing, discharge at 50 °C

**Table 4:** Reactant amounts and sample identification for the model reactions considered in this work. Reactions carried out at 140 °C for 30 min under mechanical stirring. Only the Blank sample was not subjected to reaction conditions.

Scheme ID	MFC Type	MFC Amount (g) [2 wt% in Water]	Reactant (Amount)	Histidine Amount (mg)
Blank	Exilva P-L	1	/	/
Blank-H	Exilva P-L	100	/	200
P-AGE	Exilva P-L	100	AGE (2 mL)	200
F-AGE	Exilva F-L	100	AGE (2 mL)	200
P-ESO	Exilva P-L	100	ESO (2.00 g)	200
F-ESO	Exilva F-L	100	ESO (2.00 g)	200

**Table 5:** TGA results: mass loss (%) at different temperature ranges for the analysed samples.

	Mass Loss (*w*/*w*%)			
**Sample**	T < 150 °C	150 < T < 850 °C	T = 850 °C (Air)	Residue
**Blank-H**	4.35	70.58	14.57	10.51
**P-AGE**	0.04	82.90	15.95	1.12
**P-ESO**	0.06	81.27	17.49	1.19

**Table 6:** Maximum (S′max) and minimum (S′min) torque, delta torque (ΔS′), scorch time (ts2), curing time (t90) and vulcanisation rate (t90–ts2) for the compounds reported in Table 1.

	CB N330 @140 °C	CB N330 @80 °C	CB N330 + Hist	MFC (Exilva P)	MFC (Exilva F)	MFC + Hist (Exilva P)	MFC + Hist (Exilva F)
S′max (dNm)	17.31	19.30	18.25	15.85	15.59	16.37	16.68
S′min (dNm)	0.64	0.73	0.73	0.55	0.49	0.75	0.75
ΔS′ (dNm)	16.67	18.54	17.52	15.30	15.10	15.62	15.93
ts2 (s)	110	117	118	229	206	189	194
t90 (s)	202	305	216	353	444	322	390
t90–ts2 (s)	92	188	98	124	238	133	197

**Table 7:** Main results of strain sweep tests of the compounds listed in Table 1.

	CB N330 (@140 °C)	CB N330 (@80 °C)	CB N330 + Hist	MFC (Exilva P)	MFC (Exilva F)	MFC + Hist (Exilva P)	MFC + Hist (Exilva F)
G*max (0.1% strain) [kPa]	2434	3207	2802	881	1012	946	1044
G*min (100% strain) [kPa]	634	648	665	522	459	519	530
Payne effect (ΔG*)	1799	2582	2137	359	553	427	513
G′max (0.1% strain) [kPa]	2411	3207	2781	879	1010	944	1042
G′min (100% strain) [kPa]	622	636	651	499	454	508	526
ΔG′ (G′_max_–G′_min_)	1789	2571	2130	380	556	436	516
G″min [kPa]	124	126	134	33	51	30	42
G″max [kPa]	375	605	470	82	148	84	129
tan delta min	0.137	0.123	0.122	0.064	0.076	0.055	0.041
tan delta max	0.290	0.358	0.310	0.144	0.249	0.136	0.208

**Table 8:** Tensile tests results: elongation at break (εB, %), modulus at break (MB, MPa), modulus at 100% strain (M100, MPa), modulus at 300% strain (M300, MPa), stiffness (ratio between modulus at 300% strain and modulus at 100% strain, M300/M100), and Hardness (Shore A) values, reported with their standard deviations (average of five specimens).

	εB (%)	MB (MPa)	M100	M300	M300/M100	Hardness (Shore A)
CB N330 (@140 °C)	560 ± 37	21.1 ± 0.7	1.7 ± 0.1	7.7 ± 0.7	4.6 ± 0.2	57.3 ± 0.9
CB N330 (@80 °C)	444 ± 13	27.2 ± 0.2	3.5 ± 0.1	16.7 ± 0.8	4.8 ± 0.1	63.7 ± 0.9
CB N330 + hist	495 ± 21	19.3 ± 1.5	2.0 ± 0.2	9.0 ± 0.7	4.5 ± 0.1	63.2 ± 0.3
MFC (Exilva F)	395 ± 20	10.1 ± 0.7	4.1 ± 0.3	7.4 ± 0.2	1.9 ± 0.1	62.8 ± 0.4
MFC + hist (Exilva F)	468 ± 19	9.6 ± 0.4	2.7 ± 0.1	5.0 ± 0.1	1.9 ± 0.1	61.8 ± 0.6
MFC (Exilva P)	387 ± 15	9.8 ± 0.6	4.1 ± 0.3	7.5 ± 0.3	1.8 ± 0.1	61.8 ± 0.9
MFC + hist (Exilva P)	441 ± 18	7.7 ± 0.9	2.0 ± 0.2	4.5 ± 0.1	1.7 ± 0.1	63.7 ± 0.7

## Data Availability

The data supporting the findings of this study are available from the corresponding author upon reasonable request.

## Abbreviations

The following abbreviations are used in this manuscript:

MFC	Microfibrillated cellulose
ENR	Epoxidised natural rubber
CB	Carbon black
ESO	Epoxidised soybean oil
AGE	Allyl glycidyl ether
NR	Natural rubber
SBR	Styrene–butadiene rubber
NBR	Nitrile–butadiene rubber
HNBR	Hydrogenised nitrile–butadiene rubber
